# Association between hyperuricemia and all-cause mortality in people taken Statins: a retrospective cohort study

**DOI:** 10.3389/fphar.2025.1533709

**Published:** 2025-02-19

**Authors:** Bin Zhu, Zijun He, Mingfen Wu, Jiping Huo, Zhigang Zhao, Wei Guo, Li Yang

**Affiliations:** ^1^ Department of Pharmacy, Beijing Tiantan Hospital, Capital Medical University, Beijing, China; ^2^ Neurointerventional Center, Beijing Neurosurgical Institute and Beijing Tiantan Hospital, Capital University, Beijing, China; ^3^ Beijing Hospital of Traditional Chinese Medicine, Capital Medical University, Beijing, China

**Keywords:** Statin (HMG-CoA reductase inhibitor), hyperuricemia, all-cause mortality, National Health and Nutrition Examination Survey, serum uric acid

## Abstract

**Background:**

Statins are one of the most widely prescribed medicines in clinical practice. Their benefits have extended beyond cardiovascular applications to reduce serum uric acid levels. This study aims to investigate the relationship of hyperuricemia with the risk of all-cause mortality among individuals taking statins.

**Method:**

A retrospective cohort study was performed using data from the National Health and Nutrition Examination Survey (NHANES) database between 2005 and 2018. The weighted Cox proportional hazards models were used to investigate the relationship between hyperuricemia and all cause-mortality.

**Results:**

A total of 1,958 participants were enrolled for analysis. Of them 1,429 participants were with normal uric acid levels and 529 people were with hyperuricemia. After 12 years of follow-up, there were 267 participants who died from all-cause mortality in the non-hyperuricemia group and 136 died in the hyperuricemia group. Additionally, 32.49% of participants took more than five kinds of medicines in the non-hyperuricemia group compared to 42.05% participants in the hyperuricemia group. Even after adjusting for confounding factors, we found that the serum uric acid (SUA) level was significantly correlated with all-cause mortality among statin users (HR = 1.13, 95% CI:1.02–1.24, p = 0.0161). Additionally, hyperuricemia resulted in significant increases in all-cause mortality relative to non-hyperuricemia participants in three models (HR = 1.51, 95% CI:1.16–1.96, P = 0.0023).

**Conclusion:**

Although statins have been shown to reduce uric acid levels, hyperuricemia is still significantly associated with the all-cause mortality in people taking statins. Those taking statins and having hyperuricemia should pay special attention to their SUA level.

## Introduction

Serum uric acid (SUA) is a crucial substance that exerts a substantial influence on metabolism and various physiological processes (Wang et al., 2024). Elevated SUA concentrations, as a metabolite of ingested or endogenous guanine and adenine, have been linked to an increased risk of all-cause mortality (Li et al., 2023; Yang et al., 2023). Epidemiological evidence has suggested that various metabolic conditions, including hypertension, diabetes, obesity, and metabolic syndrome, are associated with elevated SUA levels (Crawley et al., 2022).

Hyperuricemia is caused by an increase in the concentration of SUA, which is defined as SUA levels of ≥7 mg/dL in men and ≥6 mg/dL in women (Ren et al., 2024). It is estimated that approximately 38 million adults or 16.9% of the population in the United States are affected by hyperuricemia (Leung et al., 2022). As an important worldwide public health issue, hyperuricemia is reported to be associated with long-term mortality (Yin et al., 2024).

Statins, which are also known as 3-hydroxy-3-methylglutaryl-coenzyme A (HMG-CoA) reductase inhibitors, are the most broadly prescribed medical regimens to reduce low-density lipoprotein (Khatiwada and Hong, 2024). Due to their pleiotropic effects on physiological function, they are used for the primary prevention of cardiovascular disease in adults (Paparodis et al., 2024). High-quality trials showed that statin therapy was associated with a reduced risk of all-cause and cardiovascular mortality, as well as cardiovascular disease (CVD) events, even in people over the age of 75 years (Chou et al., 2022; Orkaby et al., 2020). Despite these profound effects, statins are renowned for their ability to reduce SUA levels, especially for atorvastatin and simvastatin (Akbari et al., 2024).

Although accumulating evidence reveals that hyperuricemia is associated with an increased risk of all-cause mortality, no studies have yet examined such a relationship among people taking statins. Considering the much-needed survival benefit by statins, it is quite necessary to make further exploration. In this cohort study, we sought to evaluate the relationship of hyperuricemia with the risk of all-cause mortality among individuals taking statins in a large cohort by using the National Health and Nutrition Examination Survey (NHANES) data.

## Materials and methods

### Study population

All original data enrolled for analyses were collected from the NHANES database (https://www.cdc.gov/nchs/nhanes/index.htm), which is a publicly available database that includes the results of a nationally representative survey that assesses the health and nutrition status of the US civilian population at different time periods. A total of seven periods, namely, 2005–2006, 2007–2008, 2009–2010, 2011–2012, 2013–2014, 2015–2016, and 2017–2018, are used in the study.

Participants who completed the questionnaire “Prescription Medications” were enrolled in the analyses for further screening. Participants who have taken statins are enrolled for analysis, regardless of the type of statins. Those with missing data including the key laboratory index, smoking and drinking status, and survival status were excluded. All-cause mortality data were drawn from the National Center for Health Statistics (https://www.cdc.gov/nchs/data-linkage/mortality-public.htm). The NCHS Research Ethics Review Board approved all NHANES protocols of the survey (https://www.cdc.gov/nchs/nhanes/irba98.htm). All individual privacy is kept strictly confidential, and all anonymous data were exclusively used for academic research.

### Definition and assessment of variables

Baseline characteristics including laboratory parameters were extracted from the database year by year. The diagnostic criteria of hyperuricemia were defined as SUA ≥6.0 mg/dL in female individuals and ≥7.0 mg/dL in male individuals. Triglycerides (TG) were classified as follows: less than 150 mg/dL (normal), 150–199 mg/dL (elevated), 200–499 mg/dL (high), and 500 mg/dL or above (very high) (Miller et al., 2011). Total cholesterol levels were classified as normal (less than 200 mg/dL), borderline high (200–239 mg/dL), and high (over 240 mg/dL) (He et al., 2021). Glucose levels were determined according to WHO criteria, with a cutoff point of 126 mg/dL. Drinking was defined as either normal or heavy alcohol use, according to the definition of the National Institute on Alcohol Abuse and Alcoholism, with men drinking more than five standard cups and women drinking more than four standard cups on any given day categorized as heavy alcohol use (https://www.niaaa.nih.gov/alcohol-health/overview-alcohol-consumption/moderate-binge-drinking). Smoking statuses were defined using the following question: Do you now smoke cigarettes?

### Statistical analysis

All analyses were performed using the statistical package R (version 4.2) and Empower (Empower XYS 6.0PC Version, X&Y Solutions, Inc., Boston, MA). According to the Centers for Disease Control and Prevention guidelines (https://wwwn.cdc.gov/nchs/nhanes/tutorials/default.aspx), the sample weight was assigned to each person participating in NHANES; therefore, the proposed weighting methodology was used in our analysis. Continuous variables were presented as weighted means and 95% CI, while categorical variables were described as weighted percentages (95% CI). Baseline characteristics were compared using Pearson’s chi-squared tests (categorical variables) or weighted linear regression (continuous variables). The Cox proportional hazard function model was used to investigate the association between hyperuricemia and all cause-mortality. Confounding factors including age, gender, BMI, smoking, drinking, ethnicity, the number of taken medicines, days of statin administration, direct HDL cholesterol, total cholesterol, glucose, creatinine, and triglycerides were adjusted in different models. The overall survival time was illustrated using a Kaplan–Meier curve and compared with a log-rank test with confounding factors adjusted. A two-tailed *p*-value < 0.05 was considered statistically significant.

## Results

### Basic characteristics

**FIGURE 1:**
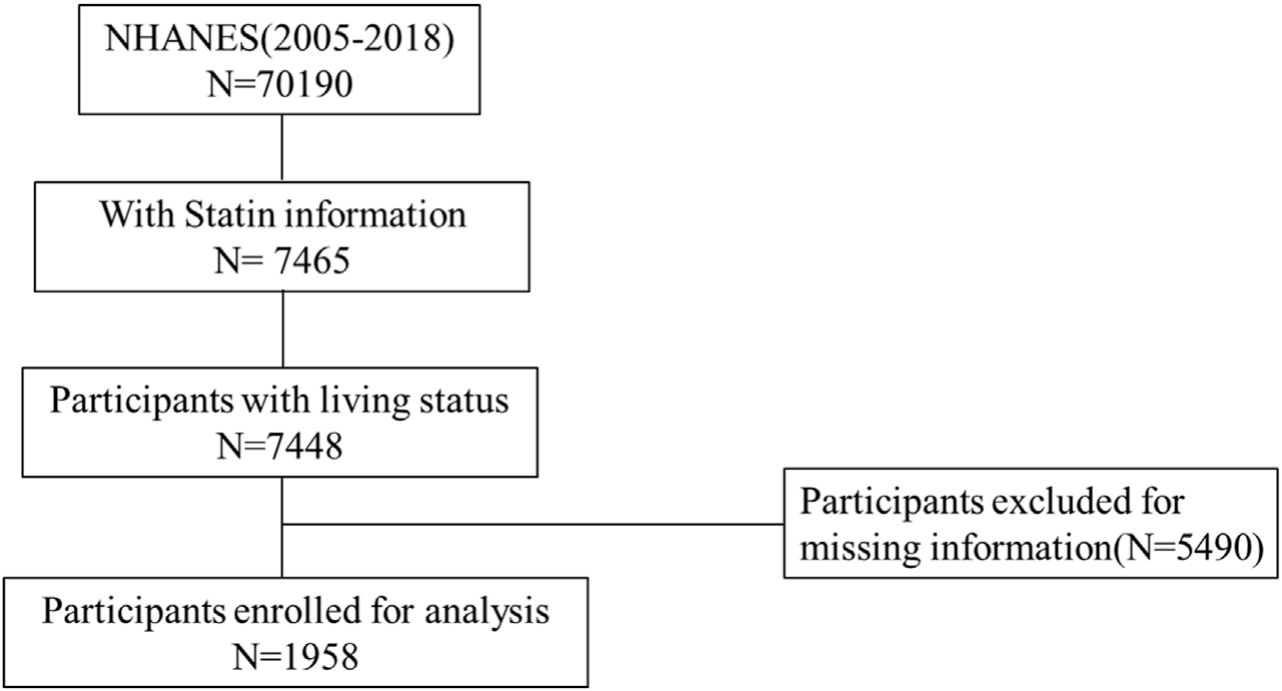
Flowchart of participants’ selection.

The database contains 70,190 participants from 2005 to 2018. Of them, 7,465 had completed the “Prescription Medications” questionnaire and provided the names of statins. After excluding the participants with missing data, 1,958 people were left for further analysis. The selection process is illustrated in Figure 1. A total of 1,429 participants had normal uric acid levels, while 529 people had hyperuricemia. The average age in the non-hyperuricemia group is 62.32 years, while the hyperuricemia group averages 64.25 years.

**FIGURE 2:**
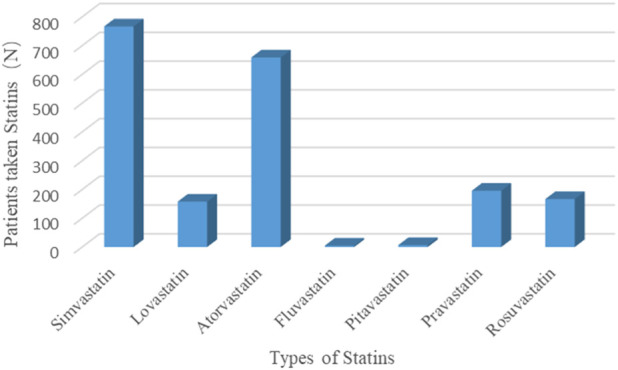
Different types of statins in our enrolled study.

There were 267 participants who died from of all-cause mortality in the non-hyperuricemia group and 136 people in the hyperuricemia group. Additionally, 907 participants in the non-hyperuricemia group took less than five kinds of medicines compared to 277 participants in the hyperuricemia group. There were 92 participants in the non-hyperuricemia group who had taken more than 10 kinds of medicines compared to 48 participants in the hyperuricemia group. The detailed basic demographic characteristics are shown in Table 1. Seven kinds of statins, namely, simvastatin, lovastatin, atorvastatin, fluvastatin, pitavastatin, pravastatin, and rosuvastatin, were found among these participants (Figure 2).

**TABLE 1:** Baseline characteristics of the enrolled participants. Data are presented as the weighted mean (95% CI) for continuous variables or weighted percentage (95% CI) for categorical variables.

	N	Non-hyperuricemia HR (95%CI)	N	Hyperuricemia HR (95%CI)	*p*-value
Age	1,429	62.32 (61.58, 63.06)	529	64.25 (63.05, 65.44)	0.003
BMI (kg/m^2^)	1,429	29.63 (29.14, 30.11)	529	32.45 (31.67, 33.23)	<0.0001
Gender					0.0890
Male	994	65.01 (61.22, 68.62)	326	59.24 (53.67, 64.58)	
Female	435	34.99 (31.38, 38.78)	203	40.76 (35.42, 46.33)	
Ethnicity					0.022
Mexican American	154	3.91 (2.99, 5.10)	33	2.19 (1.42, 3.38)	
Other Hispanic	108	2.78 (2.12, 3.64)	31	2.00 (1.26, 3.15)	
Non-Hispanic White	842	82.27 (79.71, 84.57)	307	80.68 (75.71, 84.84)	
Non-Hispanic Black	232	6.51 (5.31, 7.96)	125	9.74 (7.41, 12.72)	
Other race	93	4.53 (3.39, 6.02)	33	5.38 (3.10, 9.19)	
Smoking					0.017
Every day	330	23.28 (20.29, 26.57)	127	19.85 (15.95, 24.42)	
Some days	74	5.06 (3.59, 7.08)	15	2.27 (1.21, 4.19)	
Not at all	1,025	71.66 (68.62, 74.51)	387	77.89 (73.31, 81.87)	
Drinking					0.642
Normal alcohol use	1,282	89.33 (86.83, 91.41)	464	90.19 (86.56, 92.92)	
Heavy alcohol use	147	10.67 (8.59, 13.17)	65	9.81 (7.08, 13.44)	
All-cause mortality					
Alive	1,162	86.00 (83.91, 87.86)	393	77.16 (72.29, 81.38)	
Death	267	14.00 (12.14, 16.09)	136	22.84 (18.62, 27.71)	
Direct HDL cholesterol (mg/dL)	1,429	53.10 (51.76, 54.45)	529	49.99 (48.32, 51.66)	0.0074
Triglycerides (mg/dL)	1,249	165.07 (156.20, 173.94)	529	188.53 (171.52, 205.54)	0.017
LDL cholesterol (mg/dL)	1,429	177.60 (174.78, 180.43)	529	178.86 (173.86, 183.86)	0.669
Glucose (mg/dL)	1,429	111.55 (108.53, 114.56)	529	113.92 (109.09, 118.74)	0.429
Creatinine (mg/dL)	1,429	0.93 (0.90, 0.95)	529	1.10 (1.07, 1.13)	<0.0001
Uric acid (mg/dL)	1,429	5.21 (5.14, 5.27)	529	7.52 (7.42, 7.63)	<0.0001
Number of medicines taken					0.0024
≤5	907	67.50 (64.73, 70.16)	277	57.95 (52.19, 63.50)	
5–10	430	27 (24.56, 29.59)	204	33.39 (28.20, 39.02)	
>10	92	5.49 (3.98, 7.54)	48	8.66 (5.95, 12.45)	
Days of statins					0.4300
Tertile 1	383	25.51 (22.46, 28.82)	127	21.84 (17.83, 26.45)	
Tertile 2	545	37.80 (34.53, 41.19)	219	39.87 (34.75, 45.23)	
Tertile 3	501	36.69 (33.33, 40.18)	183	38.29 (32.22, 44.75)	

For continuous variables: weighted mean (95% CI), *p*-value was calculated by weighted linear regression. For categorical variables: weighted percentage (95% CI), *p*-value was calculated by the weighted chi-squared test.

### Associations of hyperuricemia with all-cause mortality

**FIGURE 3:**
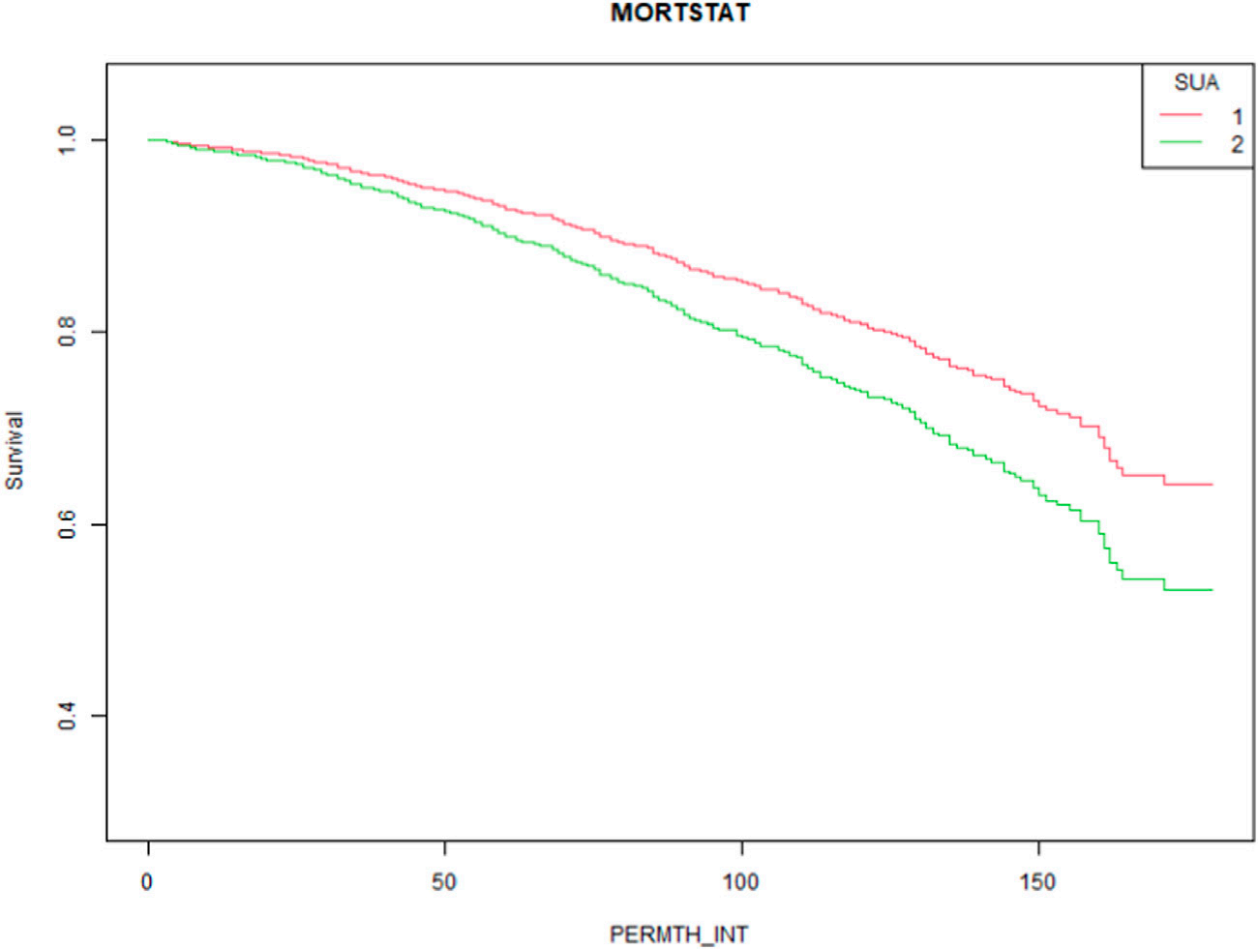
Kaplan–Meier survival curves for all-cause mortality in patients taking statins complicated with normal uric acid (1) and hyperuricemia (2).

To elucidate the relationship between hyperuricemia and all-cause mortality in people taking statins, we constructed three models using the Cox proportional hazard function model. The HRs and 95% CIs for these three models are listed in Table 2. Our results show that the SUA level was significantly correlated with the all-cause mortality in the crude model (HR = 1.18, 95% CI:1.06–1.31, P = 0.0028). Even after controlling for confounding factors, the difference was still significant (HR = 1.13, 95% CI:1.02–1.24, and p = 0.0161). Additionally, participants were further divided into hyperuricemia and non-hyperuricemia groups. Compared with the non-hyperuricemia group, hyperuricemia caused significant increases in all-cause mortality (HR = 1.71, 95% CI:1.29–2.27, P = 0.0002) in the crude model. This trend is still significant even after adjusting the confounding factors (HR = 1.51, 95% CI:1.16–1.96, P = 0.0023). The Kaplan–Meier curve is shown in Figure 3 (P = 0.0016).

**TABLE 2:** Associations between hyperuricemia and the risk of all-cause mortality in participants who have taken statins.

	HR (95% CI)					
	Model 1	*p*-value	Model 2	*p*-value	Model 3	*p*-value
SUA level	1.18 (1.06–1.31)	0.0028	1.16 (1.04–1.29)	0.006	1.14 (1.03–1.26)	0.011
Hyperuricemia						
No	Reference		Reference		Reference	
Yes	1.71 (1.29–2.27)	0.0002	1.66 (1.26–2.18)	0.0003	1.56 (1.18–2.07)	0.002

Model 1: Crude model.

Model 2: Adjusted for Age, gender, BMI, smoking, drinking, and Ethnicity.

Model 3: Adjusted for age, gender, BMI, smoking, drinking, ethnicity, the number of prescription medicines taken, days of statin administration, direct HDL cholesterol, total cholesterol, glucose, creatinine, and triglycerides.

### Stratified analysis of hyperuricemia and all-cause mortality

We evaluated the interaction of various variables and the relationship between hyperuricemia and all-cause mortality (Table 3). The results revealed that there were no significant interactions found for age, BMI, drinking, smoking, gender, total medicine taken, days of statin administration, triglycerides, glucose, and total cholesterol. Stratified analysis of hyperuricemia and all-cause mortality by other variables in statin users can be found in the Supplementary Materials.

**TABLE 3:** Stratified analysis of hyperuricemia and all-cause mortality.

	N	HR (95%CI)	*p*-value	*p*-value for interaction
BMI				
<30	1,057	1.5 (1.2, 2.1)	0.003	0.835
≥30	901	1.6 (1.2, 2.2)	0.002	
Drinking				0.217
Normal	1746	1.2 (1.1, 1.3)	<0.001	
Heavy	212	1.1 (0.9, 1.4)	0.414	
Smoking				0.139
Every day	457	1.3 (0.8, 2.0)	0.316	
Some days	89	4.0 (1.4, 11.4)	0.009	
Never	1,412	1.5 (1.2, 1.9)	0.001	
Gender				0.0768
Male	1,320	1.3 (1.0, 1.7)	0.026	
Female	638	2.0 (1.4, 2.9)	<0.001	
Total medicines taken				0.121
≤5	1,184	1.0 (0.7, 1.5)	0.807	
5–10	634	1.7 (1.2, 2.3)	<0.001	
>10	140	1.3 (0.7, 2.3)	0.387	
Days of statins				0.177
Tertile 1	510	1.6 (1.1, 2.5)	0.018	
Tertile 2	764	1.2 (0.8, 1.6)	0.360	
Tertile 3	685	1.8 (1.3, 2.6)	<0.001	
Triglycerides				0.273
<150	1,095	1.4 (1.1, 1.9)	0.016	
150–200	360	1.4 (0.8, 2.4)	0.196	
200–500	463	1.9 (1.2, 2.8)	0.004	
≥500	40	5.8 (1.0, 34.6)	0.055	
Glucose				0.904
<126	1,534	1.5 (1.2, 1.9)	0.001	
≥126	424	1.4 (1.0, 2.1)	0.086	
Total cholesterol				0.542
<200	1,480	1.5 (1.2, 1.9)	<0.001	
200–240	351	1.3 (0.8, 2.2)	0.297	
≥240	127	2.2 (0.9, 5.3)	0.083	
Age				0.996
<60	563	1.4 (0.7, 2.8)	0.276	
≥60	1,395	1.5 (1.2, 1.8)	<0.001	

## Discussion

In a comprehensive analysis using the NHANES database, we evaluated the relationship between hyperuricemia and all-cause mortality in people taking statins. Our results revealed that hyperuricemia is significantly associated with the all-cause mortality in people taking statins. This trend still existed even after we adjusted the cofounding factors. To the best of our knowledge, our study is the first to assess the relationship between hyperuricemia and all-cause mortality in people taking statins.

Hyperuricemia, one of the serious health problems for individuals with gout and cardiovascular diseases, is a metabolic disorder resulting from prolonged impaired elimination of uric acid (Prabhakar and Lopez-Candales, 2024; Du L et al., 2024). Data from observational cohort studies and meta-analysis suggest that hyperuricemia is associated with all-cause mortality (Zuo et al., 2016; Han et al., 2023; Rao et al., 2024). In addition, Keller et al. found that statin use was associated with a lower risk of mortality in gout patients (Keller et al., 2018). However, no study has observed whether hyperuricemia is associated with a much higher risk of all-cause mortality in people taking statins. Thus, to address the knowledge gaps, we conducted this retrospective cohort study. We believe these findings warrant extra consideration, especially for people who have taken statins and have hyperuricemia.

Statins are one of the most widely prescribed medicines due to their ability to reduce the risk of cardiovascular and cerebrovascular diseases (Mangione et al., 2022; Lavie et al., 2021; Gan et al., 2024). Studies have revealed that it is not only anti-inflammatory but also aids in plaque stabilization (Cesaro et al., 2024). In addition, a recent meta-analysis found that statins can reduce serum uric acid levels, adding to the body of evidence supporting statins’ role in protecting people dying from CVD (Akbari et al., 2024; Derosa et al., 2016; Athyros et al., 2004). The mechanisms linking statins to lower SUA levels are still not fully elucidated. As SUA may play a role in the formation of free radicals and oxidative stress, we speculate that this effect may be associated with the anti-oxidative and anti-inflammatory effects (Sorensen et al., 2019; Athyros et al., 2004).

Although we observed a relationship between hyperuricemia and all-cause mortality, there are several limitations that should also be noted. First, we only observed all-cause mortality in people taking stains. As we know, statins mainly play a role in preventing cardiovascular and cerebrovascular events; nevertheless, the all-cause mortality can be influenced by a lot of factors. Thus, the results of this study should be carefully evaluated in a large cohort. Second, the limited sample size limits the importance of our research. Although the total number of participants in the NHANES was large, participants with missing variables that were incorporated in the analysis were removed from the study, resulting in a significantly decreased number of enrolled participants. Third, the retrospective design and reliance on observational data limit the ability to draw causal conclusions. In addition, some selection bias cannot be excluded from the analysis. Thus, the discovery of our findings warrants further validation in future clinical practice.

## Conclusion

Statins are widely used in the prevention of cardiovascular and cerebrovascular diseases. Recent studies showed that statins can also help in reducing SUA levels. Our study revealed that hyperuricemia is significantly associated with the all-cause mortality in people taking statins. The findings hold significant clinical implications, emphasizing the importance of patients taking statins being aware of the potential negative impact of hyperuricemia. However, due to the bias caused by the small sample size, future studies in a large cohort are warranted to verify the conclusion.

## Data Availability

The original contributions presented in the study are included in the article/Supplementary Material; further inquiries can be directed to the corresponding authors.
